# Exploring the Association Between Lipid-Lowering Medications and Dry Eye Disease in COVID-19 Patients

**DOI:** 10.22336/rjo.2025.82

**Published:** 2025

**Authors:** Konstantin Yaakov Gushansky, Pinja Sutinen, Sohee Jeon, Raimo Tuuminen

**Affiliations:** 1 Department of Ophthalmology, Shaare Zedek Medical Center, Jerusalem, Israel; 2 Helsinki Retina Research Group, Faculty of Medicine, University of Helsinki, Helsinki, Finland; 3 Keye Eye Center, 326 Teheran-ro, Gangnam-gu, Seoul, South Korea; 4 Faculty of Health Sciences, Ben-Gurion University of the Negev, Beer-Sheva, Israel; 5 Department of Ophthalmology, Kymenlaakso Central Hospital, Kotka, Finland

**Keywords:** COVID-19, dry eye disease, HMG-CoA reductase inhibitors, statins, SARS-CoV-2, systemic medication, DED = Dry eye disease, OR = Odds ratio, COVID-19 = Coronavirus disease 2019, SARS-CoV-2 = Severe acute respiratory syndrome coronavirus 2, HMG-CoA = 3-Hydroxy-3-methylglutaryl-CoA, ICU = Intensive care unit

## Abstract

**Purpose:**

To explore the associations between systemic lipid-lowering medications and dry eye disease (DED) in patients hospitalized for SARS-CoV-2.

**Methods:**

This retrospective cohort study analyzed electronic medical records of SARS-CoV-2 patients hospitalized at Shaare Zedek Medical Center from April 1, 2020, to December 31, 2024. Adults without a previous DED diagnosis who had an ophthalmologic examination confirming no DED within the year preceding hospitalization were included. Exclusions included ICU admissions, specific systemic conditions, ocular surgeries, and systemic medications known to induce DED. Patients were categorized into those who developed DED within six months post-hospitalization and those who did not.

**Results:**

Among the 1,165 patients, 167 (14.3%) developed DED post-hospitalization. After adjusting for age, gender, and SARS-CoV-2 vaccination status, an inverse association between statins and dry eye disease was evident. Treatment with any statin was associated with reduced odds of developing dry eye disease (OR 0.289, p<0.001), particularly with atorvastatin (OR 0.374, p<0.001) and simvastatin (OR 0.297, p<0.001).

**Discussion:**

The inverse association observed in this study supports a potential protective effect of statins, consistent with their known anti-inflammatory properties and their ability to reduce cholesterol-driven Meibomian gland dysfunction.

**Conclusion:**

Statin use is inversely associated with the development of DED in SARS-CoV-2 hospitalized patients. The anti-inflammatory and cholesterol-lowering effects of statins provide a robust framework for understanding the underlying pathophysiological mechanisms.

## Introduction

Dry eye disease (DED) is a common chronic condition that causes discomfort, visual disturbances, and impairs the quality of life [**1,2**]. DED is a multifactorial condition that can be caused or aggravated by systemic diseases or oral medications [**1,3**]. SARS-CoV-2 is one of the newly recognized systemic diseases that cause DED. It has been reported that 10-40% of the individuals affected by SARS-CoV-2 are expected to suffer from ocular symptoms, most commonly DED [**4-7**]. Although the precise pathophysiology remains to be elucidated, SARS-CoV-2 inoculation and subsequent inflammatory changes in the ocular conjunctiva [**8**] and meibomian glands [**6**] have been proposed as underlying mechanisms of DED resulting from SARS-CoV-2 infection.

The impact of lipid-lowering drugs on DED has been highly controversial; several studies implicated statins as risk factors for DED [**9-11**], while other studies have suggested they are protective against DED [**12-17**]. Among lipid-lowering drugs, HMG-CoA reductase inhibitors (statins) have pleiotropic effects, including suppressing inflammation, reducing oxidative stress, and inhibiting T-cell activation, in addition to lowering endogenous cholesterol biosynthesis [**18,19**].

We aim to fill this gap by exploring potential associations between lipid-lowering drugs and DED incidence among hospitalized patients with SARS-CoV-2.

## Methods

This retrospective cohort study used electronic medical records of patients admitted to Shaare Zedek Medical Center in Jerusalem, Israel, for SARS-CoV-2 infection from April 1, 2020, to December 31, 2024. All medical care, including hospitalization, outpatient follow-up, and ophthalmologic diagnoses, was provided exclusively by Shaare Zedek Medical Center to the patients in our study cohort. The report adhered to the ethical principles outlined in the Declaration of Helsinki, as amended in 2013. The Institutional Review Board waived the need for ethics approval and obtaining consent for the collection, analysis, and publication of retrospectively obtained and anonymized data for this non-interventional study (document #48755).

### Diagnosis of DED

Study participants were selected from individuals who were definitively negative for all the following diagnostic criteria for DED:

1. Subjective symptoms such as ocular discomfort, irritation, or foreign body sensation;

2. Tear function abnormalities, defined as a tear breakup time (TBUT) ≤ 5 seconds or a Schirmer test result ≤ 5 mm in 5 minutes;

3. Positive vital staining, demonstrated by fluorescein staining (or, less commonly, lissamine green or rose bengal).

At the end of the follow-up period, a diagnosis of DED was established only in participants who met at least two of the above criteria.

### Study population

Eligible participants were adults aged 18 years or older with no prior diagnosis of DED, who were hospitalized at Shaare Zedek Medical Center for a minimum of 24 hours due to acute SARS-CoV-2 infection. Participants were selected from individuals who had undergone at least one ophthalmologic examination at our hospital within the preceding 12 months. Only those with a complete ocular history and examination sufficient to confidently exclude DED, as defined in the previous section, were included.

Exclusion criteria encompassed admission to an intensive care unit; diagnoses of Sjogren’s syndrome, other autoimmune or rheumatologic disorders; malignancy; neurological conditions such as cerebrovascular accident or Parkinson’s disease; history of topical ocular medication, contact lenses, or ocular surgery during the preceding 24 months; any corneal refractive surgery, ocular trauma, or history of chemical exposure. Additionally, any individuals receiving chemotherapy or initiating chronic medications known to induce DED during the preceding 12 months of acute SARS-CoV-2 infection were excluded. These medications included anticholinergics, anxiolytics, antipsychotics, antidepressants, oral steroids, beta-blockers, analgesics, hormonal treatments, and diuretics [**3,9,20**].

Exclusion criteria also applied to participants who did not have an additional ophthalmic checkup within 12 months following hospital discharge, as well as to those with an ophthalmic follow-up period of less than 6 months or incomplete follow-up. Patients who had changes in chronic medications during follow-up were also excluded from the study.

We categorized the SARS-CoV-2 cohort into two groups: patients who developed DED within 6 months of hospitalization and those who did not.

### Observation procedures

Diagnosis of DED was determined from ophthalmologists’ reports and ICD-10 diagnoses. All research participants have undergone DED work-up, including: thorough history on DED symptoms; examination of the face, eyelids, lacrimal system, and ocular surface; tear break-up time; corneal and conjunctival fluorescein staining; fluorescein dye disappearance test; and corneal sensation, Schirmer’s test, and laboratory work-up for Sjogren disease, according to clinical judgement.

Reports with DED diagnosis and mention of either pertinent ophthalmic findings (e.g., superficial punctate keratopathy, short tear break-up time), ocular dryness in free text, or ocular lubricant recommendation/prescription, were considered positive for DED. Patients with severe DED requiring intervention such as punctal occlusion or tarsorrhaphy were referred to an oculoplastic surgeon. In SARS-CoV-2 patients, the incidence of DED was compared between patients treated with a given medication and those who were not.

### Statistical analysis

For two-group comparisons, Student’s t-test was employed for continuous variables, while proportions underwent the χ^2^ test with Yates’ correction. Logistic regression was utilized for multivariate analysis, incorporating age, gender, and vaccination status as covariates. Data analysis was performed using SPSS version 27 (SPSS Inc., Chicago, IL). P-values less than 0.05 were deemed statistically significant.

## Results

### Baseline variables

The cohort comprised 1165 patients, with 167 (14.3%) diagnosed with DED following hospitalization and 998 (85.7%) without a DED diagnosis. The mean follow-up period was 26.4 months (range 6.0-37.1). Patients with and without DED exhibited comparable characteristics regarding age, gender distribution, vaccination status, and the length of hospitalization (**Table 1**).

**Table 1:** Baseline characteristics of SARS-CoV-2 hospitalized patients

Study cohort	DED - (N=998)	DED + (N=167)	
Age (y)	59.1 ± 18.4	59.0 ± 16.5	0.947
Females (%)	460 (46.1%)	77 (46.1%)	1.000
Hospitalization length (d)	3.48 ± 8.74	3.61 ± 7.75	0.857
Vaccinated (%)	547 (54.8%)	91 (54.5%)	0.936

Data are given as mean ± SD or absolute numbers and proportions. For two-group comparisons, qualitative data were analyzed with the two-factor χ^2^ with Yates’ correction and continuous variables with the Student’s T-test. DED = dry eye disease; Vaccinated = received at least one vaccine dose.

We assessed the incidence of DED across different lipid-lowering drugs. Among patients with at least one lipid-lowering medication (either a statin or a triglyceride-lowering drug), DED was observed in 29 of 455 (6.4%) patients. In contrast, among patients without such medication, DED was observed in 138 of 710 (19.4%) patients (p<0.001, **Table 2**). Moreover, among patients with DED, the average number of lipid-lowering drugs was significantly lower (0.19±0.43) compared to those without DED (0.46±0.56, p<0.001).

**Table 2:** Number of chronic medications among 1165 patients hospitalized for SARS-CoV-2

	DED - (N=998)	DED + (N=167)	p-value
Number of all drug prescriptions	3.88 ± 2.65	3.90 ± 5.80	0.941
Number of lipid-lowering drugs	0.46 ± 0.56	0.19 ± 0.43	**<0.001**
At least one lipid-lowering drug	426 (42.7%)	29 (17.4%)	**<0.001**

Data are given as mean ± SD or absolute numbers and percentages. For two-group comparisons, qualitative data were analyzed with the two-factor χ^2^ with Yates’ correction and continuous variables with the Student’s T-test. P-value indicated in bold is less than 0.05.

### Statins were associated with less dry eye disease

Next, we sub-analyzed the prevalence of DED to understand whether there is a significant difference in the prevalence of DED according to the action mechanism of each drug. In our cohort, patients treated with any statin exhibited significantly lower DED incidence than those without statin treatment (6.5% vs. 19.3%, respectively; p<0.001, **Table 3**). Treatment with atorvastatin, simvastatin, and rosuvastatin was associated with significantly lower DED incidence compared to lack of treatment with these medications (7.8% vs. 16.6%, p<0.001; 6.4% vs. 15.2%, p=0.013; and 0% vs. 14.9%, respectively, p=0.008, **Table 3**).

**Table 3:** Distribution of 167 dry eye disease cases regarding lipid-lowering medications among 1165 SARS-CoV-2 hospitalized patients

Drug (N= -/+)	Number (prevalence) of patients with DED		p-value
Statins	Drug -	Drug +	
Any statin (716/449)	138 (19.3%)	29 (6.5%)	**<0.001**
Atorvastatin (869/296)	144 (16.6%)	23 (7.8%)	**<0.001**
Simvastatin (1056/109)	160 (15.2%)	7 (6.4%)	**0.013**
Rosuvastatin (1124/41)	167 (14.9%)	0 (0%)	**0.008**
Pravastatin (1160/5)	167 (14.4%)	0 (0%)	0.358
Sandostatin (1164/1)	167 (14.4%)	0 (0%)	0.682
**Triglyceride-lowering drugs**			
Any triglyceride-lowering (1130/35)	166 (14.7%)	1 (2.9%)	**0.048**
Ezetimibe (1139/26)	166 (14.6%)	1 (3.9%)	0.124
Bezafibrate (1157/8)	167 (14.4%)	0 (0%)	0.246
Ciprofibrate (1164/1)	167 (14.4%)	0 (0%)	0.682

Data are given as absolute numbers and proportions of patients with dry eye disease. For two-group comparisons, qualitative data were analyzed using the two-factor χ^2^ test with Yates’ correction. DED = dry eye disease. Bold for p-value less than 0.05.

Among patients treated with triglyceride-lowering drugs, a significantly lower prevalence of DED was observed with any triglyceride-lowering medication compared with those not on treatment (2.9% vs. 14.7%, p=0.048, **Table 3**). Of the 35 patients with triglyceride-lowering drugs, 29 were on concomitant statin medication. When triglyceride-lowering drugs were adjusted with statin medication, no significant association with DED remained (p=0.083).

### Multivariate analysis

After adjusting for potential confounders, patients’ age, gender, and vaccination status, an inverse association between lipid-lowering drugs and DED persisted. Any statin treatment reduced the odds of developing DED (OR 0.289; 95% CI 0.190-0.439, p<0.001, **Table 4**). Specifically, atorvastatin and simvastatin had reduced odds for the development of DED (OR 0.374; 95% CI 0.234-0.596, p<0.001, and OR 0.297; 95% CI 0.135-0.655, p<0.001, respectively, **Table 4**).

**Table 4:** Logistic regression analysis adjusted for age, gender, and vaccination status among 1165 SARS-CoV-2 hospitalized patients

		Odds Ratio	95% CI	p-value
Demographics	Age	1.000	(0.991, 1.009)	0.971
	Female gender	0.988	(0.707, 1.381)	0.944
Vaccination	Vaccinated	1.028	(0.733, 1.441)	0.872
Polypharmacy		1.629	(1.099, 2.416)	**0.015**
	Any statin	0.289	(0.190, 0.439)	**<0.001**
Statins	Atorvastatin	0.374	(0.234, 0.596)	**<0.001**
	Simvastatin	0.297	(0.135, 0.655)	**<0.001**
Triglyceride-lowering drugs		0.171	(0.023, 1.259)	0.083

CI = confidence interval. Bold for p-value less than 0.05. Polypharmacy: chronic treatment with five or more medications.

## Discussion

The etiology of DED is intricate and multifaceted. This is particularly important among patients hospitalized for SARS-CoV-2, who exhibit various pathological changes in the ocular surface, such as reduced lipid layer thickness and shorter tear film break-up time (BUT), and are thus at higher risk for DED [**6**]. While some medication groups, e.g., anticholinergics and diuretics, have been linked to DED [**1,3**], they account for only a fraction of the cases. In our pursuit of identifying additional risk factors, we have deliberately excluded these potential confounders from our study.

A population with SARS-CoV-2 infection was selected as the ideal candidate for our study for several key reasons. First, it is widespread and continues to affect a growing population across all demographics, unlike diseases such as Alzheimer’s, which primarily impact those with specific risk factors. Second, COVID-19 patients have exhibited a higher prevalence of DED (~10% prevalence of DED), nearly double that of the general population (~6% in the general U.S. population). Third, SARS-CoV-2 infection is typically an acute, self-limiting disease that rarely imposes long-term impacts on patients’ lives post-recovery, unlike conditions such as stroke or hip fractures. This reduces the likelihood of significant lifestyle changes that could introduce confounding factors into the study. Fourth, SARS-CoV-2 infection, unless resulting in ICU treatment, seldom necessitates starting or modifying medication regimens, as is common with conditions like myocardial infarction (MI). This helps minimize confounding from medication changes. Finally, hospitalized COVID-19 patients received extensive medical attention and regular checkups, ensuring reliable data collection. Our pilot study may open the door to novel therapeutic options to alleviate the burden of dry eye disease among the expanding population of SARS-CoV-2 survivors after hospitalization. We firmly believe that the findings presented in this manuscript significantly contribute to the existing knowledge on dry eye disease and its associated risk factors.

Multiple studies have documented ophthalmic manifestations, some severe, in patients with SARS-CoV-2 infection [**21,22**] and, rarely, after vaccination [**23**]. We observed that most ocular manifestations occurred in the anterior segment of the eye, with dry eye disease being the most clinically relevant association with SARS-CoV-2 infection, and thus warrants further study. Here, we focused solely on DED. In literature, ocular symptoms, e.g., dry eye disease and conjunctivitis, are relatively common after SARS-CoV-2 infection and may appear even before the onset of respiratory symptoms [**24**]. One-third of patients with SARS-CoV-2 infection had ocular abnormalities, which were more common in those with more severe disease [**25**]. Ocular surface manifestations were the most common. Dry eye, itching, and foreign-body sensations were present in 60% of individuals experiencing ocular problems [**26**]. Given the high incidence of ocular surface manifestations, the protective effect of statin therapy against DED among hospitalized patients with SARS-CoV-2 infection warrants further attention.

Within our cohort, several statins, indicating the drug class effect, were associated with reduced acute DED among SARS-CoV-2 survivors. We found an inverse association between atorvastatin and simvastatin, which are predominantly lipophilic statins, and DED. Statins are known to minimize Meibomian gland dysfunction (MGD) [**12**] and blepharitis [**13**], both of which contribute to DED [**14,15**]. Two pathological models have been proposed to explain the protective effect of statins on the Meibomian gland: inflammatory and meibum instability.

Inflammatory cytokines such as IL-1, IL-6, IL-17, and IFN-γ are elevated in blepharitis and DED [**27,28**]. Statins, known for their cholesterol-independent anti-inflammatory and immunomodulatory effects, downregulate T helper cells and decrease the secretion of those cytokines [**29,30**]. In the human eye, lipophilic statins were associated with, e.g., lower MMP-2 levels [**31**]. This might alleviate disruption of corneal barrier function, a component in the pathophysiology of DED.

The meibum, an oily substance that prevents tear evaporation, was found to have increased cholesterol ester levels in MGD [**11**]. This further leads to a higher melting point, increased viscosity, and a propensity toward Meibomian gland obstruction [**11**]. Thus, MGD induces tear evaporation, ocular inflammation, and increased tear film osmolarity, all of which are components of evaporative DED [**16,32-34**]. Statins could help prevent MGD by inhibiting cholesterol production in all eyelid sebaceous glands, thereby decreasing meibum viscosity and minimizing Meibomian gland blockage [**35**]. This explains the improvement of blepharitis scores in patients treated with topical ocular atorvastatin [**17**]. Notably, the treatment also improved tear film BUT and conjunctival congestion, which are significant components of DED [**17**].

We also found an inverse association between triglyceride-lowering medications and DED. Increased triglyceride levels are associated with MGD [**36,37**] and DED [**32**]. The underlying mechanism is thought to involve a higher melting point and lower viscosity of meibum, as well as a propensity to block Meibomian gland orifices in patients with hypertriglyceridemia [**38**]. Nevertheless, a vast majority (83%) of patients with triglyceride-lowering medication were on concomitant statin treatment. After adjusting for statin treatment, triglyceride-lowering medication remained non-significant regarding the existence of DED. We established a positive correlation between the higher number of chronic oral medications and DED. A statistically significant correlation between polypharmacy and DED existed, particularly among patients with five or more oral medications, where polypharmacy was a risk factor increasing the odds for DED by 62.9%. These findings are consistent with previous research linking polypharmacy to DED [**39,40**].

While it is challenging to attribute the emergence of DED within six months of hospital discharge solely to HMG-CoA reductase inhibitor use, given that lifestyle changes may also play a role, our study examined the statistical correlations between HMG-CoA reductase inhibitor use and DED. Significant associations with drugs were observed as a protective factor against DED, emphasizing that the medications themselves may be influencing the development of DED. These findings would warrant further investigation into an RCT approach into the specific medication subgroup, offering valuable insights into how these medications could contribute to or prevent DED. Such findings could ultimately help inform preventative strategies for dry eye disease.

Several limitations should be acknowledged in our study. Although our selection criteria focused on SARS-CoV-2 inpatients and excluded intensive care unit hospitalizations, we did not directly assess SARS-CoV-2 severity in our study cohort. The cohort consisted of patients hospitalized for acute SARS-CoV-2 infection lasting at least 24 hours. Exclusion criteria encompassed admission to an intensive care unit. These selection criteria aimed to exclude mild-to-moderate and extreme cases of SARS-CoV-2 infection and to reduce variability in SARS-CoV-2 infection severity. Unfortunately, there was no further grading of infection severity. To date, grading scales for the infection are rough, and no meaningful grading systems are available to assess its severity in non-ICU hospitalized patients. The entire cohort of patients was comprised of survivors of SARS-CoV-2 infection, leaving the effect of SARS-CoV-2 infection itself on DED beyond the scope of the study design. Statin medication can be regarded as a surrogate marker for lipid and vascular conditions that may affect dry eye disease, and including both variables - the disease itself and the medication for it - would lead to the alpha error in multivariate analysis since these factors are strongly bonded with each other and thus cannot be regarded as independent variables.

Furthermore, the retrospective design of our study inherently limited the scope of our findings. There were no uniform criteria for diagnosing DED or for quantifying its severity due to the study design. The study was therefore diagnosis-based, relying on the uniform ICD-10 diagnosis codes. These findings, however, warrant further prospective randomized controlled trials analyzing corneal fluorescence staining (CFS) score, Schirmer scores, tear break-up time (TBUT), and meibomian gland dysfunction (MGD) following statin use. The study also did not include long-term data on the protective effects of statins or on the progression and management of DED in these patients, as these data were not consistently available in the medical records. Patients were not routinely followed up for more than 6 months unless they had long COVID or other specific symptoms that warranted further follow-up. It is challenging to determine whether a given lipid-lowering medication is protective against the development of DED or whether confounding factors, such as dyslipidemia, drug-to-drug interactions, or additional systemic medications, are at play. Moreover, the results of this study cannot be generalized to a population with a history of topical ocular medication or other measures or conditions that were a part of the exclusion criteria for this study.

Nevertheless, the inverse associations found between lipid-lowering medications and DED could open novel therapeutic options for SARS-CoV-2 inpatients experiencing DED. Such studies hold the potential to alleviate the burden of DED within the expanding population of SARS-CoV-2 survivors after hospitalizations.

## Conclusion

In conclusion, our pilot study uncovers novel associations between several statins and DED. We anticipate that these insights will enrich current understanding of DED and potentially improve its management.
